# Activation of mitophagy antagonizes high uric acid–induced hepatic lipid accumulation

**DOI:** 10.1016/j.jbc.2025.111054

**Published:** 2025-12-13

**Authors:** Jiayu Chen, Hairong Zhao, Yuemei Xi, Shanpan Fu, De Xie, Linqian Yu, Qiang Wang, Binyang Chen, Qian Zhang, Mingyan Zhang, Xueling Ye, Mengni Wu, Wanling Que, Shuyi Chen, Yayan Liu, Tetsuya Yamamoto, Hidenori Koyama, Hong Zhao, Jidong Cheng

**Affiliations:** 1 Department of Endocrinology, Xiang’an Hospital of Xiamen University, School of Medicine, Xiamen University, Xiamen, Fujian, China; 2 Yunnan Provincial Key Laboratory of Entomological Biopharmaceutical R&D, College of Pharmacy, Dali University, Dali, Yunnan, PR China; 3 Department of Health Evaluation Center, Osaka Gyoumeikan Hospital, Osaka, Japan; 4 Department of Diabetes, Endocrinology and Clinical Immunology, Hyogo College of Medicine, Nishinomiya, Hyogo, Japan; 5 Xiamen Key Laboratory of Translational Medicine for Nucleic Acid Metabolism and Regulation, Xiang’an Hospital of Xiamen University, School of Medicine, Xiamen University, Xiamen, Fujian, China

**Keywords:** hepatic lipid accumulation, hyperuricemia, mitophagy, CD36, PINK1–Parkin pathway

## Abstract

The rising prevalence of hyperuricemia associated with lifestyle changes has been confirmed as an independent risk factor for metabolic dysfunction–associated fatty liver disease. Mitochondria, as central regulators of lipid metabolism, maintain functional homeostasis through mitophagy, the selective removal of damaged or dysfunctional mitochondria. However, whether and how high uric acid (HUA) induces mitophagy and the mechanistic role of mitophagy in hyperuricemia-induced hepatic lipid metabolism disorders remain to be elucidated. Our study demonstrated that HUA induces hepatic fat accumulation and damaging mitochondria in primary mouse hepatocytes. Simultaneously, mitophagy was activated by HUA, evidenced by upregulated expression and phosphorylation of PINK1 and Parkin, enhanced LC3B-I to LC3B-II conversion, and enhanced TOM20–LC3B immunofluorescence colocalization. In urate oxidase gene knockout (*Uox*-KO) mice (a model of sustained hyperuricemia), we detected significantly elevated expression of mitophagy-related proteins in liver tissues, accompanied by marked lipid accumulation and inflammatory responses. Further studies demonstrated that HUA upregulates CD36 protein expression. CD36 knockdown alleviated lipid accumulation in primary mouse hepatocytes, whereas PINK1 knockdown exacerbated this effect through further CD36 upregulation. Notably, treatment with the mitophagy activator urolithin A significantly ameliorated hepatic lipid accumulation and inflammation in *Uox*-KO mice. These findings demonstrate that the PINK1–Parkin-mediated mitophagy activated by HUA serves as a protective mechanism against HUA-induced hepatic fat accumulation. Our results suggest that mitophagy regulation may represent a novel therapeutic target for HUA-induced hepatic fat accumulation.

Uric acid (UA) is the end product of purine metabolism in the human body (1). Hyperuricemia is diagnosed when excessive UA production or impaired excretion results in a serum uric acid (SUA) level ≥0.42 mmol/l (7 mg/dl) (2). In recent years, the prevalence of hyperuricemia has increased significantly, largely because of changes in lifestyle and diet (3). As a common metabolic disorder, hyperuricemia is pathophysiologically associated with the development of various metabolic-related diseases (4), including insulin resistance (5), obesity (6), cardiovascular diseases (7), kidney diseases (8), and liver diseases (9).

Metabolism-associated fatty liver disease (MAFLD) is the most prevalent chronic liver disease worldwide (10), primarily characterized by excessive hepatic lipid accumulation (11). The global prevalence of MAFLD is estimated at approximately 32.4%, with a notably higher incidence in men (39.7%) than in women (25.6%), and it continues to rise (12). Increasing evidence indicates a strong association between hyperuricemia and the pathogenesis of MAFLD (13). Notably, commonly prescribed UA-lowering agents have shown potential therapeutic benefits in improving MAFLD outcomes (14). Furthermore, the SUA-to-creatinine ratio has emerged as a valuable biomarker for liver disease evaluation and is now widely applied in clinical practice (15).

Hepatic lipid accumulation is a hallmark pathological feature of MAFLD and is tightly regulated by a dynamic balance among four key metabolic processes: free fatty acid (FFA) uptake, *de novo* lipogenesis (DNL), fatty acid oxidation, and lipid efflux (16). These interconnected pathways work in concert to maintain hepatic lipid homeostasis. In short-term hepatic lipid metabolism, 59.0% ± 9.9% of hepatic fatty acids originate from circulating FFAs, 26.1% ± 6.7% from DNL, and 14.9% ± 7.0% from dietary intake (17). Disruption in the regulation of these processes leads to metabolic reprogramming, a central mechanism driving the progression of MAFLD.

Mitochondria are highly dynamic organelles that play a central role in cellular energy metabolism (18). Mitophagy, a selective form of macroautophagy, is essential for maintaining mitochondrial homeostasis by removing damaged or excess mitochondria (19). The PINK1–Parkin signaling pathway is a common mitophagy regulatory mechanism (20). Emerging evidence indicates that high uric acid (HUA) modulates mitophagy in a tissue-specific manner—promoting mitophagy in renal and cardiac tissues, whereas suppressing it in endothelial cells (21, 22, 23). However, the effects of HUA on hepatic mitophagy and its underlying regulatory mechanisms remain poorly understood. Our previous research demonstrated that HUA activates hepatic macroautophagy (24); nonetheless, its precise role in mitophagy regulation within hepatocytes has yet to be elucidated.

Mitochondria are central to hepatic lipid metabolism (25). Under conditions of high-fat diet (HFD)–induced hepatic lipid accumulation, mitophagy is notably suppressed in hepatocytes (26). In MAFLD patients, mitochondrial fatty acid oxidation capacity is significantly impaired, accompanied by downregulation of key markers associated with mitochondrial dynamics, including biogenesis, autophagy, fission, and fusion (27). Despite these insights, the specific alterations in mitophagy and its functional role in HUA-induced hepatic lipid accumulation remain largely unexplored.

In this study, we utilized a human hepatoblastoma cell line (HepG2), primary mouse hepatocytes, and a uricase knockout (*Uox*-KO) mouse model to investigate whether and how HUA induces mitophagy in hepatocytes. Furthermore, we explored the regulatory role of mitophagy in HUA-induced hepatic lipid accumulation, aiming to provide novel mechanistic insights into the pathogenesis of hyperuricemia-associated metabolic dysfunction.

## Results

### HUA induced hepatic fat accumulation

**Figure 1:**
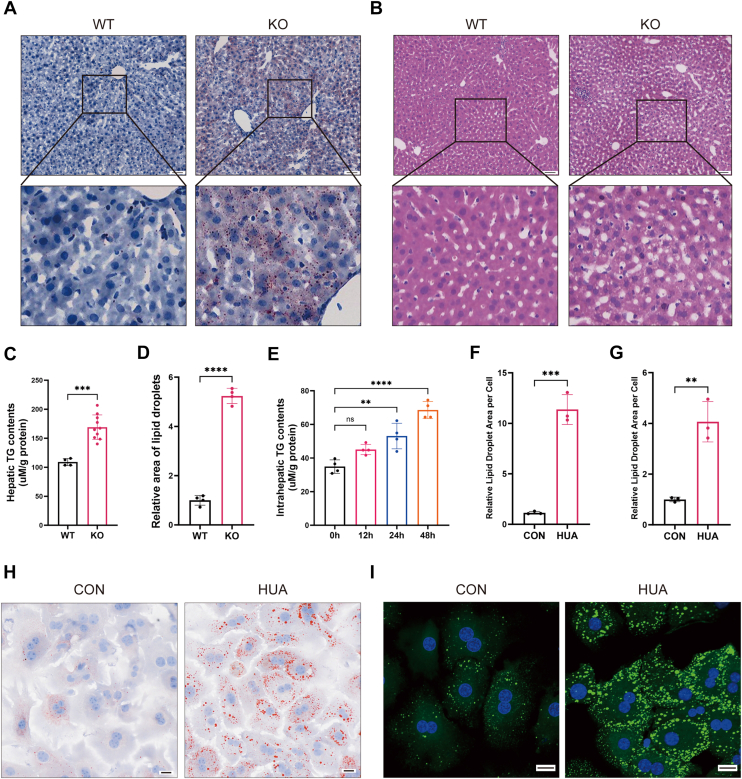
**High uric acid (HUA) induces hepatic fat accumulation**. *A*, representative images of Oil Red O staining in liver sections from WT and *Uox*-KO mice and (*D*) quantification of the Oil Red O staining. The scale bar represents 50 μm. *B*, representative images of H&E staining in liver sections from WT and *Uox*-KO mice. The scale bar represents 50 μm. *C*, biochemical analysis of hepatic triglyceride (TG) in WT and *Uox*-KO mice (n = 4–8 per group). *E*, measurement of intracellular TG content in primary mouse hepatocytes following HUA (15 mg/dl) stimulation at different time points (0, 12, 24, and 48 h; n = 4). *H*, representative images of Oil Red O staining of primary mouse hepatocytes after 48 h of HUA stimulation and (*F*) quantification of the Oil Red O staining. The scale bar represents 20 μm. *I*, representative images of neutral lipid staining with Bodipy 493/503 (*green*) of primary mouse hepatocytes after 48 h of HUA stimulation and (*G*) quantitative analysis of green fluorescence. Nuclei were stained with DAPI (*blue*), and merged images of both labeling are shown. The scale bar represents 20 μm. Individual data points on the graph represent data from each mouse or each independent experiment. Data are presented as mean ± SD. Statistical analysis was done using unpaired *t* test and one-way ANOVA, Tukey’s post hoc analysis. ∗*p* < 0.05, ∗∗*p* < 0.01, ∗∗∗*p* < 0.001, and ∗∗∗∗*p* < 0.0001 compared with the indicated groups. DAPI, 4',6-diamidino-2-phenylindole.

To clarify the effect of HUA on hepatic fat accumulation, we utilized a *Uox*-KO mouse model characterized by consistently elevated SUA levels for our experiments (28). Oil Red O and H&E staining were performed to assess hepatic lipid accumulation in mice. Compared with WT mice, *Uox*-KO mice exhibited substantial lipid droplet deposition and tissue vacuolization in liver tissues (Fig. 1, *A* and *B*, *D*), along with a significant increase in hepatic triglyceride (TG) content (Fig. 1*C*). In addition, *Uox*-KO mice showed elevated fasting blood glucose, serum TG, serum total cholesterol (TC), low-density lipoprotein cholesterol, and serum alanine aminotransferase (ALT) levels (Table1). We further conducted experiments in primary mouse hepatocytes and found that, compared with the control group, HUA stimulation time-dependently increased intracellular TG content (Fig. 1*E*). This finding was corroborated by both Oil Red O staining (Fig. 1, *F* and *H*) and BODIPY staining (Fig. 1, *G* and *I*).

**Table 1:** Body weight and biochemical profiles of WT and *Uox*-KO mice

Parameters	WT (n = 4)	KO (n = 10)
Body weight, g	22.10 ± 0.84	22.30 ± 1.48
Serum UA, μmol/l	82.46 ± 14.47	370.50 ± 52.91^a^
Fasting blood glucose, mmol/l	3.48 ± 0.21	4.62 ± 0.64^b^
Serum TG, mmol/l	0.42 ± 0.01	0.57 ± 0.10^c^
Serum TC, mmol/l	2.25 ± 0.43	3.15 ± 0.27^d^
Serum high-density lipoprotein cholesterol, mmol/l	2.90 ± 0.28	2.45 ± 0.81
Serum LDL-C, mmol/l	1.41 ± 0.19	2.66 ± 0.88^c^
Serum ALT, U/l	21.46 ± 1.84	28.67 ± 1.87^a^
Serum aspartate aminotransferase, U/l	17.08 ± 2.65	17.19 ± 1.67
Hepatic TC, μM/g protein	50.46 ± 3.65	50.18 ± 5.05

Data are presented as mean ± SD. Statistical analysis was done using unpaired *t* test.

c *p* < 0.05.

b *p* < 0.01.

d *p* < 0.001.

a *p* < 0.0001 compared with the indicated groups.

### HUA induces mitochondrial damage in hepatocytes

**Figure 2:**
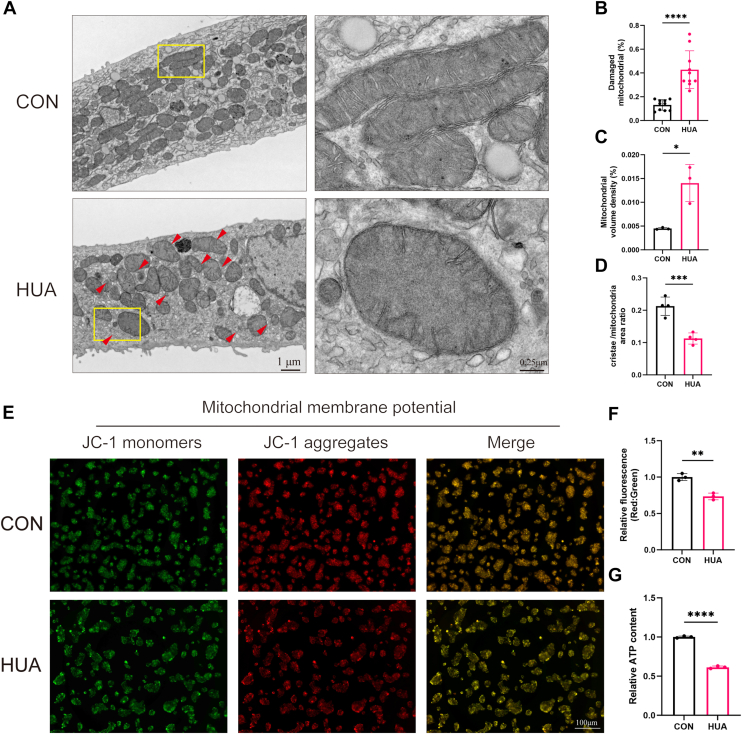
**High uric acid (HUA) induces mitochondrial damage in hepatocytes**. *A*, representative transmission electron microscopy (TEM) images of primary mouse hepatocytes from control (untreated) and HUA (48 h treatment) groups. *Red arrows* indicate damaged mitochondria. The scale bar represents 1 μm. *B*, quantification shown as percentage of damaged mitochondria in respect to total number. (n = 10). *C*, quantification of mitochondrial volume density (% mitochondrial area/cytosolic area)/cell and (*D*) cristae density (expressed as cristae area per mitochondrial area). *E*, mitochondrial membrane potential (MMP, ΔΨm) in HepG2 cells using JC-1 staining after 48 h of HUA exposure was measured using a JC-1 assay kit and observed using fluorescence microscopy (the scale bar represents 100 μm). *F*, JC-1 *red* light/*green* light relative fluorescence intensity analysis. *G*, the relative ATP content in primary mouse hepatocytes after 48 h of HUA stimulation was determined by measuring the fluorescence emitted from the reaction between ATP and luciferin, catalyzed by luciferase. Individual data points represent each independent experiment or a random field of view. Data are presented as mean ± SD. Statistical analysis was done using unpaired *t* test. ∗∗∗∗*p* < 0.0001 compared with the indicated groups.

Mitochondria, as key organelles in hepatic lipid metabolism, play a dynamic role in maintaining the balance between lipid synthesis and catabolism (29). To investigate the effects of HUA on hepatocyte mitochondria, primary mouse hepatocytes were treated with HUA. Transmission electron microscopy analysis revealed that, compared with the control group, HUA-treated hepatocytes exhibited a marked increase in damaged mitochondria (evidenced by heightened mitochondrial swelling and reduced cristae density) (Fig. 2, *A*–*D*).

Loss of mitochondrial membrane potential (MMP) is a hallmark of mitochondrial dysfunction. To assess the impact of HUA on MMP, we employed the JC-1 fluorescent probe, which emits red fluorescence in healthy mitochondria but shifts to green fluorescence when MMP is depolarized. Our results demonstrated that HUA exposure resulted in a lower red fluorescence intensity and a lower red/green ratio compared with the control group (Fig. 2, *E* and *F*). These findings indicate a substantial loss of MMP, further confirming mitochondrial impairment following HUA exposure. In addition, we assessed ATP production in primary mouse hepatocytes using chemiluminescence assays. The results showed that HUA decreased ATP production in primary mouse hepatocytes (Fig. 2*G*).

### HUA induces mitophagy in hepatocytes

**Figure 3:**
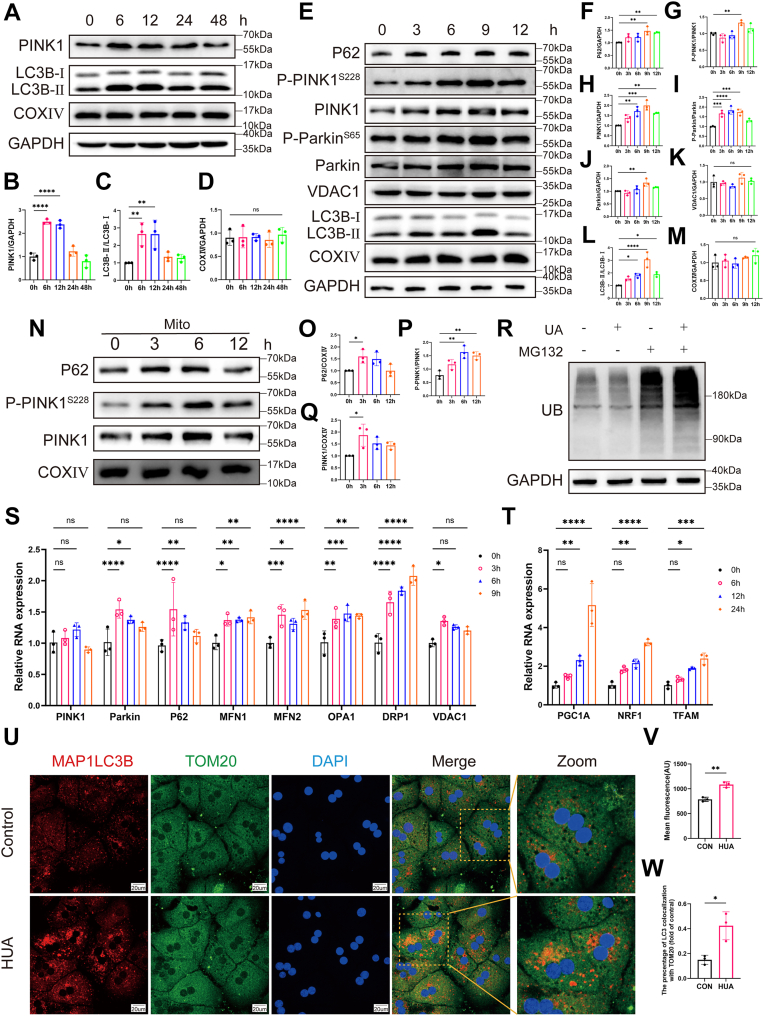
**High uric acid (HUA) induces mitophagy in hepatocytes**. *A*, representative Western blot (WB) images of PINK1, LC3B, and COXⅣ protein expression in primary mouse hepatocytes treated with HUA for different durations (0, 6, 12, 24, and 48 h). *B*–*D*, quantification of PINK1, LC3B-II/I, and COXⅣ protein levels in primary hepatocytes (n = 3). *E*, representative WB images of P62, P-PINK1^S228^, PINK1, P-Parkin^S65^, Parkin, VDAC1, LC3B, and COXⅣ protein expression in primary mouse hepatocytes treated with HUA for different durations (0, 3, 6, 9, and 12 h). *F*–*M*, quantification of P62, P-PINK1^S228^, PINK1, P-Parkin^S65^, Parkin, VDAC1, LC3B-II/I, and COXⅣ protein levels in primary hepatocytes (n = 3). *N*, representative WB images of P62, P-PINK1^S228^, and PINK1 protein levels in the mitochondrial fraction of HepG2 cells following HUA treatment at various time points (0, 3, 6, and 12 h). *O*–*Q*, quantification of mitochondrial P62, P-PINK1, and PINK1 protein levels in HepG2 cells (n = 3). *R*, representative WB images of UB protein expression in primary mouse hepatocytes treated with or without HUA and MG132 (10 μM) for 9 h. *S*, RT–PCR analysis of P62, PINK1, Parkin, MFN1, MFN2, OPA1, DRP1, and VDAC1 mRNA expression in primary hepatocytes treated with HUA for different durations (0, 3, 6, and 9 h). *T*, RT–PCR analysis of PGC1A, NRF1, and TFAM mRNA expression in primary hepatocytes treated with HUA for different durations (0, 6, 12, and 24 h). *U*, representative confocal microscopy images showing LC3B (*red*) and TOM20 (*green*) colocalization in primary hepatocytes treated with HUA for 9 h. Nuclei were counterstained with DAPI (*blue*). The scale bar represents 20 μm. *V*, quantification of LC3B mean fluorescence intensity (n = 3). *W*, Pearson’s correlation coefficient between LC3B and TOM20 from experiments shown in *U* (n = 3). Individual data points represent each independent experiment. Data are expressed as mean ± SD. Statistical analysis was done using unpaired *t* test and one-way ANOVA, Tukey’s post hoc analysis. ∗*p* < 0.05, ∗∗*p* < 0.01, ∗∗∗*p* < 0.001, and ∗∗∗∗*p* < 0.0001 compared with the indicated groups. DAPI, 4',6-diamidino-2-phenylindole.

To investigate whether HUA-induced mitochondrial damage triggers mitophagy, mouse primary hepatocytes were treated with HUA for 6, 12, 24, and 48 h. Analysis of LC3B-II/I conversion indicated that autophagic activity peaked between 6 and 12 h. Concurrently, PINK1 protein levels showed an initial rise followed by a subsequent decrease, whereas COXIV expression remained stable across all time points (Fig. 3, *A*–*D*). To more precisely delineate the temporal dynamics of mitophagy activation, mouse primary hepatocytes were exposed to HUA for 3, 6, 9, and 12 h, and key mitophagy-related markers were analyzed. Western blot (WB) analysis revealed a time-dependent upregulation in the expression and phosphorylation of multiple mitophagy-related proteins—including P62, PINK1, and Parkin—together with enhanced LC3B-I to LC3B-II conversion. These changes reached a peak at 9 h after UA treatment. In contrast, the protein levels of VDAC1 and COXIV remained unaltered throughout the time course (Fig. 3, *E*–*M*). We further performed mitochondrial fractionation in HepG2 cells, using COXIV as a mitochondrial marker, to assess translocation of mitophagy-related proteins to mitochondria. Quantitative analysis revealed significantly elevated levels of mitochondrial P-PINK1^S228^, PINK1, and P62 at 3 and 6 h after UA treatment compared with controls (Fig. 3, *N*–*O*). In addition, in primary hepatocytes, total ubiquitin protein levels were increased following 9 h of UA stimulation (Fig. 3*R*), consistent with the activation of ubiquitin-dependent mitophagic machinery.

At the transcriptional level, quantitative real-time PCR (qPCR) analysis demonstrated a significant increase in Parkin and P62 mRNA levels following HUA treatment, whereas PINK1 mRNA remained unchanged, suggesting potential post-transcriptional regulation (Fig. 3*S*). Concurrent transcriptional changes were observed in key regulators of mitochondrial dynamics, including MFN1, MFN2, OPA1, and DRP1 (Fig. 3*S*). To reconcile the observed activation of mitophagy with the stable protein levels of mitochondrial loading controls, we further assessed the transcript levels of mitochondrial biogenesis–related factors. qPCR data showed that genes encoding PGC1A, NRF1, and TFAM were transcriptionally upregulated upon HUA treatment (Fig. 3*T*).

Immunofluorescence analysis further confirmed mitophagy activation, as evidenced by enhanced colocalization of LC3B (an autophagosome marker) with TOM20 (a mitochondrial marker), along with increased LC3B fluorescence intensity in HUA-treated hepatocytes (Fig. 3, *U*–*W*). Collectively, these findings confirm that HUA induces mitophagy at multiple levels, including protein expression, transcriptional regulation, and subcellular localization.

### HUA induces mitophagy in mouse liver

**Figure 4:**
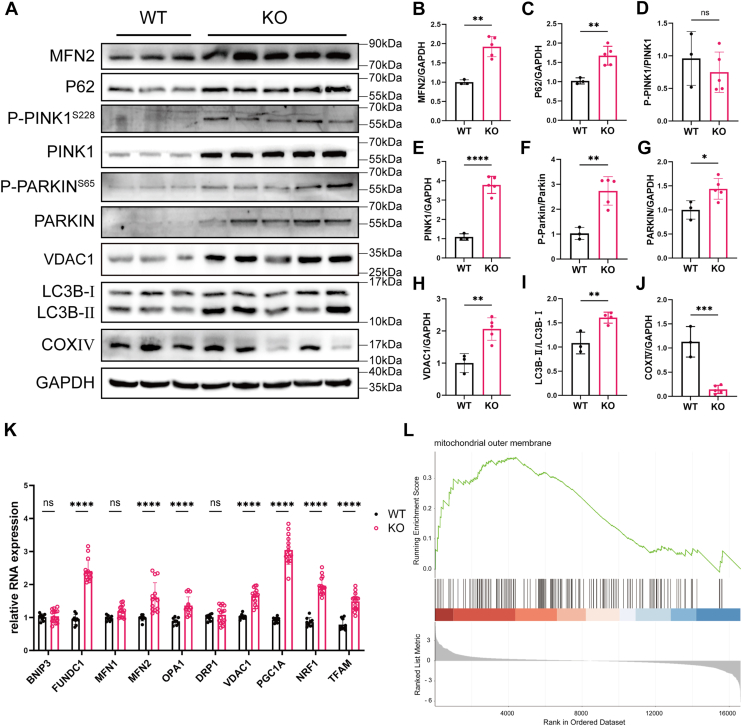
**High uric acid induces mitophagy in mouse liver**. *A*, representative Wesetrn blot (WB) images showing the expression of mitophagy-related proteins (P62, P-PINK1^S228^, PINK1, P-Parkin^S65^, Parkin, VDAC1, and LC3B-II/I), mitochondrial fusion proteins MFN2 (mitofusin 2), and the mitochondrial marker COXIV in liver tissues from WT and *Uox*-KO mice. *B*–*J*, quantification of MFN2, P62, P-PINK1^S228^, PINK1, P-Parkin^S65^, Parkin, VDAC1, LC3B-II/I, and COXIV protein levels (n = 3–5). *K*, RT–PCR analysis of mitochondrial dynamics–related gene expression in liver tissues from WT and *Uox*-KO mice (n = 4–8). *L*, gene set enrichment analysis (GSEA) plot for the “mitochondrial outer membrane” Gene Ontology term. Individual data points on the graph represent data from each mouse. Data are presented as mean ± SD. Statistical analysis was done using unpaired *t* test and one-way ANOVA, Tukey’s post hoc analysis. ∗∗*p* < 0.01, ∗∗∗*p* < 0.001, and ∗∗∗∗*p* < 0.0001 compared with the indicated groups.

To further confirm hepatic mitophagy activation in response to HUA, WB analysis of liver tissue revealed a significant increase in P62, P-PINK1^S228^, PINK1, P-Parkin^S65^, Parkin, and VDAC1 expression in *Uox*-KO mice compared with WT controls (Fig. 4, *A*, *C*–*H*). In addition, an enhanced conversion of LC3B-I to LC3B-II was observed (Fig. 4, *A* and *I*). Notably, COXIV protein expression was significantly reduced, suggesting a decrease in mitochondrial mass in *Uox*-KO mice (Fig. 4, *A* and *J*). Together, these findings strongly support the activation of mitophagy in the livers of hyperuricemic mice.

In addition, we performed qPCR to assess the expression of genes related to mitochondrial dynamics in liver tissues from WT and *Uox*-KO mice. The results showed significant upregulation of key genes involved in non–ubiquitin-dependent mitophagy (FUNDC1), mitochondrial fusion (MFN2, OPA1), and mitochondrial biogenesis (PGC1α, NRF1, and TFAM) in *Uox*-KO mice compared with WT mice (Fig. 4*F*). Consistent with the transcriptional changes, MFN2 protein expression was also elevated in *Uox*-KO liver tissues (Fig. 4, *A* and *B*). To further explore the transcriptomic alterations induced by hyperuricemia, we conducted mRNA-Seq on liver tissues from both groups. Gene set enrichment analysis revealed positive enrichment of mitochondrial outer membrane–related pathways in the *Uox*-KO mice group (Fig. 4*G*). Taken together, these results suggest that hyperuricemia not only activates mitophagy but also influences mitochondrial dynamics and enhances mitochondrial biogenesis, revealing a comprehensive adaptive response of mitochondria to UA stimulation.

### HUA upregulates CD36 expression in hepatocytes

**Figure 5:**
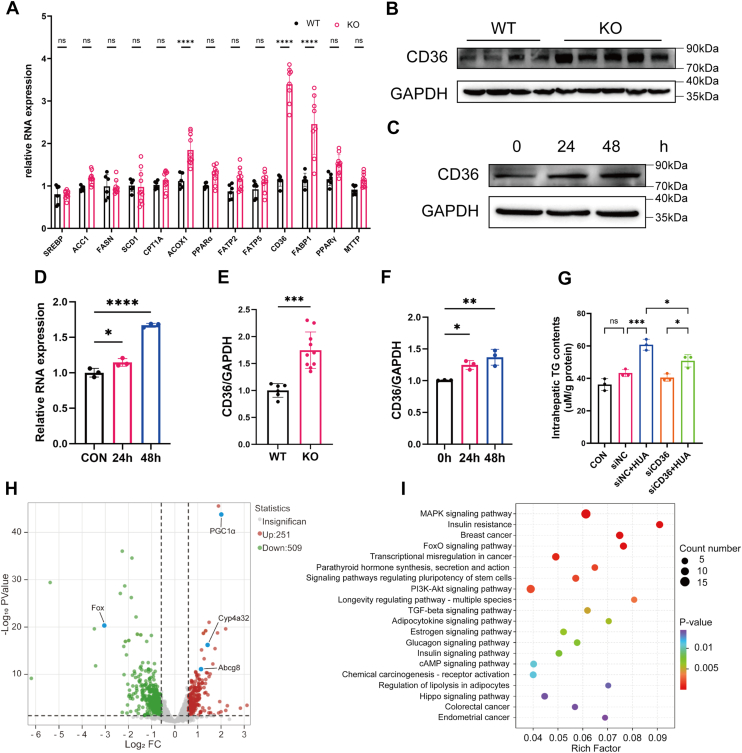
**High uric acid (HUA) upregulates CD36 expression in hepatocytes**. *A*, RT–PCR analysis of hepatic lipid metabolism–related gene expression in WT and *Uox*-KO mice (n = 4). *B*, representative Western blot (WB) images of CD36 protein expression in liver tissues from WT and *Uox*-KO mice. *C*, representative WB images of CD36 protein expression in primary mouse hepatocytes treated with HUA for different durations (0, 24, and 48 h). *D*, RT–PCR analysis of CD36 mRNA levels in primary mouse hepatocytes treated with HUA for different durations (0, 24, and 48 h; n = 3). *E* and *F*, quantification of CD36 protein levels in liver tissues (n = 4–8) and primary hepatocytes (n = 3). *G*, measurement of intracellular triglyceride (TG) content in primary hepatocytes after 48 h of HUA treatment following 6 h of siCD36 transfection (n = 3). *H*, volcano plot illustrating differentially expressed genes between *Uox*-KO and WT groups based on RNA-Seq analysis. *I*, bubble plot of KEGG enrichment analysis results in liver tissues from *Uox*-KO and WT mice. Individual data points on the graph represent data from each mouse or each independent experiment. Data are presented as mean ± SD. Statistical analysis was done using unpaired *t* test and one-way ANOVA, Tukey’s post hoc analysis. ∗*p* < 0.05, ∗∗*p* < 0.01, ∗∗∗*p* < 0.001, and ∗∗∗∗*p* < 0.0001 compared with the indicated groups. KEGG, Kyoto Encyclopedia of Genes and Genomes.

Our previous findings demonstrated that HUA contributes to hepatic lipid accumulation. In this study, we further investigated the molecular mechanisms underlying this phenomenon. qPCR analysis revealed significant upregulation of *CD36*, *FABP1*, and *ACOX1* mRNA levels in the livers of *Uox*-KO mice compared with WT controls (Fig. 5*A*). This transcriptional profile suggests a metabolic shift favoring hepatic FFA uptake in *Uox*-KO mice, characterized by enhanced FFA transport from the circulation into hepatocytes, along with compensatory activation of intracellular lipid trafficking and β-oxidation pathways. WB validation demonstrated a corresponding increase in CD36 protein expression in *Uox*-KO livers (Fig. 5, *B* and *E*). Parallel *in vitro* experiments using primary mouse hepatocytes exposed to HUA recapitulated these findings, showing upregulation of CD36 mRNA and protein expression (Fig. 5, *C* and *D*, *F*).

To determine the functional role of CD36 in HUA-induced lipid accumulation, we performed siRNA-mediated CD36 knockdown in primary hepatocytes prior to HUA exposure. CD36 silencing significantly attenuated HUA-induced TG accumulation, confirming the critical role of CD36 in UA-driven hepatic steatosis (Fig. 5*G*).

Meanwhile, the RNA-Seq analysis of liver tissue from WT and *Uox*-KO mice revealed upregulation of *PGC1α*,*Cyp4a32* and *Abcg8*, along with downregulation of the *Fox* gene in *Uox*-KO group (Fig. 5*H*). Kyoto Encyclopedia of Genes and Genomes pathway analysis of differentially expressed genes identified that the mitogen-activated protein kinase signaling pathway and insulin resistance signaling pathway were enriched (Fig. 5*I*).

### Activation of mitophagy inhibits HUA-induced upregulation of hepatic CD36 expression

**Figure 6:**
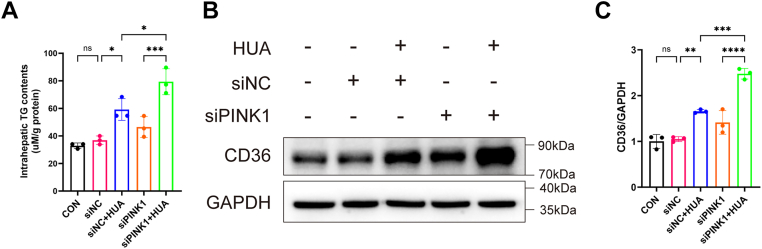
**Mitophagy plays a regulatory role in high uric acid (HUA)–induced hepatic lipid accumulation and CD36 protein expression**. *A*, measurement of intracellular triglyceride (TG) content in primary mouse hepatocytes after 48 h of HUA treatment following 6 h of siPINK1 transfection (n = 3). *B*, representative Western blot (WB) images of CD36 protein expression in primary mouse hepatocytes after 48 h of HUA treatment following 6 h of siPINK1 transfection. *C*, quantification of CD36 protein levels (n = 3). Individual data points represent each independent experiment. Data are presented as mean ± SD. Statistical analysis was done using one-way ANOVA, Tukey’s post hoc analysis. ∗*p* < 0.05, ∗∗*p* < 0.01, ∗∗∗*p* < 0.001, and ∗∗∗∗*p* < 0.0001 compared with the indicated groups.

To investigate the role of mitophagy in HUA-induced lipid accumulation, we knocked down PINK1 in primary hepatocytes using siRNA. PINK1 silencing significantly exacerbated intracellular TG accumulation and further increased CD36 protein expression under HUA stimulation (Fig. 6, *A*–*C*), indicating that impaired mitophagy promotes lipid accumulation *via* CD36 upregulation.

**Figure 7:**
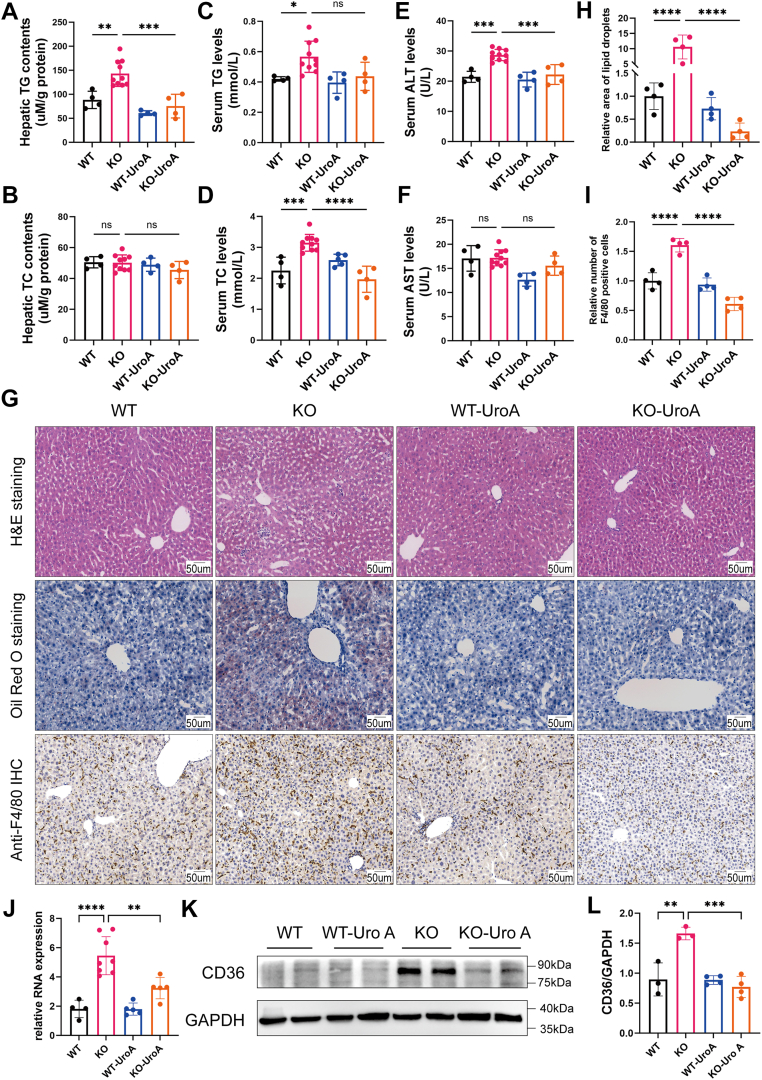
**Mitophagy activation attenuates high uric acid–induced hepatic lipid accumulation**. *A*–*F*, biochemical analysis of hepatic triglyceride (TG), total cholesterol (TC), and serum levels of TG, TC, alanine aminotransferase (ALT), and aspartate aminotransferase (AST) in WT, *Uox*-KO, WT-Uro-A, and KO-Uro-A mice (n = 4–8 per group). *G*, representative images of H&E staining, Oil Red O staining, and F4/80 immunohistochemistry in liver sections from WT, *Uox*-KO, WT-Uro-A, and KO-Uro-A mice. The scale bar represents 50 μm. *H*, quantification of hepatic lipid droplet content (n = 4). *I*, quantification of F4/80-positive cells (n = 4). *J*, RT–PCR analysis of CD36 mRNA levels in liver tissues from WT, *Uox*-KO, and KO-Uro-A mice (n = 4–8 per group). *K*, representative Wesetern blot (WB) images of CD36 protein expression in liver tissues from WT, WT-Uro-A, *Uox*-KO, and KO-Uro-A mice. *L*, quantification of CD36 protein levels (n = 4). Individual data points on the graph represent data from each mouse. Data are presented as mean ± SD. Statistical analysis was done using one-way ANOVA, Tukey’s post hoc analysis. ∗*p* < 0.05, ∗∗*p* < 0.01, ∗∗∗*p* < 0.001, and ∗∗∗∗*p* < 0.0001 compared with the indicated groups. Uro-A, urolithin A.

To validate these observations *in vivo*, we administered urolithin A (Uro-A), a known mitophagy activator, to *Uox*-KO mice and evaluated its effects on hepatic lipid metabolism. Biochemical analyses revealed significantly elevated serum TC levels and hepatic TG content in *Uox*-KO mice compared with WT mice, both of which were substantially reduced following Uro-A treatment (Fig. 7, *A* and *D*). In contrast, serum TG levels and hepatic TC content showed no significant differences among the experimental groups (Fig. 7, *B* and *C*). Histological assessment using Oil Red O staining confirmed excessive lipid deposition in *Uox*-KO mice, which was significantly reduced following Uro-A treatment (Fig. 7*H*). Liver function tests showed significantly elevated serum ALT levels in *Uox*-KO mice compared with WT controls, with Uro-A treatment effectively reducing ALT levels (Fig. 7*E*), whereas aspartate aminotransferase levels remained unchanged across all groups (Fig. 7*F*). Histopathological evaluation by H&E staining revealed characteristic punctate necrosis and inflammatory cell infiltration in the livers of *Uox*-KO mice, both of which were markedly improved following Uro-A administration (Fig. 7*G*). Furthermore, immunohistochemical analysis using F4/80 staining demonstrated increased monocyte–macrophage infiltration in *Uox*-KO mouse livers, which was significantly reduced after Uro-A treatment (Fig. 7*I*).

To further assess the regulatory relationship between mitophagy and CD36 expression, we analyzed CD36 levels in liver tissues from WT mice, WT mice treated with Uro-A, *Uox*-KO mice, and *Uox*-KO mice treated with Uro-A. WB and qPCR analyses revealed significantly elevated CD36 protein and mRNA levels in *Uox*-KO mice compared with WT controls (Fig. 7, *J*–*L*). Notably, Uro-A treatment markedly attenuated these changes, reducing hepatic CD36 mRNA levels and normalizing CD36 protein expression. Collectively, these data demonstrate that activation of mitophagy ameliorates HUA-induced hepatic lipid accumulation by downregulating CD36 expression.

## Discussion

**Figure 8:**
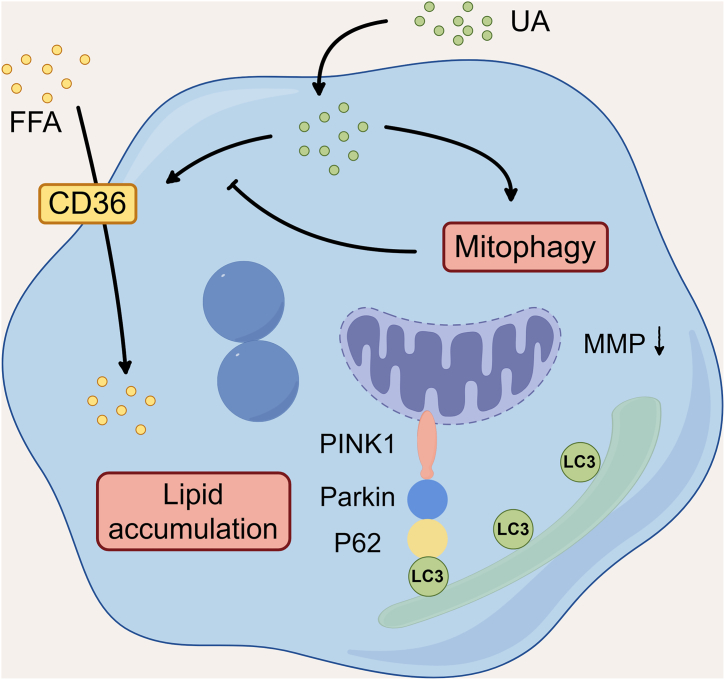
**Schematic diagram of activation of mitophagy antagonizes high uric acid–induced hepatic lipid accumulation (created with**Figdraw.com**)**. High uric acid upregulates CD36, promoting hepatic fat accumulation. Meanwhile, it activates mitophagy *via* the PINK1–Parkin pathway, which antagonizes this lipid accumulation.

With the advancement of the social economy and changes in lifestyle, the prevalence of hyperuricemia has shown a significant upward trend (3). Recent evidence suggests that elevated SUA levels are closely associated with the development and progression of MAFLD (30). Hepatic lipid accumulation, the central pathological feature of MAFLD, demonstrates a complex interplay with HUA levels (31). Indeed, a considerable number of publications have reported that hyperuricemia can induce hepatic lipid accumulation (32), and our research group's previous work further supports this conclusion (33). The findings of this study demonstrate that HUA induces hepatic lipid accumulation while concurrently impairing mitochondrial function in hepatocytes, leading to the activation of PINK1-mediated mitophagy. Further investigation revealed that the activation of mitophagy can counteract HUA-induced hepatic lipid accumulation (Fig. 8). Collectively, our research underscores the protective role of mitophagy activation in counteracting HUA-induced hepatic lipid accumulation.

The mechanisms underlying the impact of HUA on hepatic lipid metabolism remain a key focus and challenge in current research. Elevated UA levels may disrupt hepatic lipid metabolism by inducing oxidative stress (33), mitochondrial stress (34), endoplasmic reticulum stress (35), and inflammatory responses (32); however, whether additional molecular mechanisms are involved remains unclear. Previous studies have shown that HUA can activate the NLRP3 inflammasome pathway, promoting hepatic lipid accumulation and insulin resistance. Inhibition of NLRP3 expression not only significantly ameliorates abnormal hepatic lipid metabolism but also reverses HUA-induced impairment of insulin signaling (32). Another study demonstrated that HUA triggers endoplasmic reticulum stress in hepatocytes, leading to the activation and nuclear translocation of SREBP-1c, which subsequently upregulates the expression of ACC1, FAS, and SCD1, ultimately resulting in TG accumulation (35). In addition, as an end product of fructose metabolism, UA can amplify fructose-induced hepatic lipid accumulation by inducing oxidative stress and activating the NFAT5 signaling pathway, thereby increasing aldose reductase expression in HepG2 cells (36). Our previous research confirmed that HUA promotes hepatic fatty acid synthesis and lipid accumulation by inducing oxidative stress, increasing intracellular reactive oxygen species, and subsequently activating the c-Jun N-terminal kinase–activator protein-1 signaling pathway (33). Building on this foundation, the present study further investigates the molecular mechanisms underlying HUA-mediated hepatic lipid metabolic disorders. Our results demonstrate that HUA significantly upregulates CD36 expression and exacerbates hepatic lipid accumulation, whereas CD36 knockdown *via* siRNA markedly attenuates HUA-induced lipid accumulation. Notably, previous studies have shown that liver-specific CD36 knockout in HFD-fed mice significantly reduces hepatic FFA uptake and alleviates hepatic steatosis (37). Therefore, we propose that HUA promotes hepatic lipid accumulation by upregulating CD36 expression, thereby enhancing FFA uptake in hepatocytes.

Hepatic lipid metabolism is a dynamic equilibrium process primarily regulated by four key pathways: FFA uptake, DNL, fatty acid oxidation, and lipid efflux (16). Under physiological conditions, the liver maintains lipid metabolic homeostasis through precise coordination of these pathways, ensuring a balance between fatty acid supply and disposal. However, under the pathological conditions, such as HUA, this homeostasis is disrupted, leading to metabolic reprogramming (38). In this study, we found that in hyperuricemic mice, the mRNA levels of fatty acid translocase *CD36*, acyl-CoA oxidase 1 (*ACOX1*), and intracellular fatty acid–binding protein (*FABP1*) were significantly increased, whereas the expression of fatty acid synthase genes (*ACC1*, *FAS*, and *SCD1*) remained unchanged. Based on these findings, we hypothesize that in the hyperuricemia mouse model, enhanced hepatic FFA uptake leads to increased intracellular fatty acid content and transport. As a compensatory mechanism, elevated intracellular fatty acid levels may stimulate fatty acid oxidation while concurrently suppressing DNL. Notably, a stable isotope–based study in MAFLD patients revealed that hepatic fatty acid sources are derived approximately 60% from circulating FFAs, 25% from DNL, and 15% from dietary intake over the short term (17). These findings suggest that HUA may contribute to MAFLD pathogenesis through a dual mechanism: enhancing hepatic FFA uptake capacity and promoting DNL. Collectively, these processes synergistically drive the pathological progression of MAFLD.

Mitophagy, a specialized form of selective autophagy, plays a pivotal role in eliminating dysfunctional or damaged mitochondria and maintaining intracellular mitochondrial homeostasis (39). In the pathological progression of MAFLD, mitophagy acts as a critical regulatory mechanism by modulating hepatocyte energy metabolism and maintaining oxidative stress balance (26). A comparative study revealed that patients with MAFLD exhibit significantly impaired mitochondrial fatty acid oxidation capacity, along with downregulated expression of markers related to mitochondrial dynamics (including biogenesis, autophagy, fission, and fusion) compared with healthy controls (27). Notably, studies have reported an inverse correlation between hepatic lipid content and mitophagy rates in HFD-fed mice. Liver-specific knockout of PARKIN led to impaired mitophagy and exacerbated hepatic steatosis, inflammation, and fibrosis (40). Recent evidence also highlights the therapeutic potential of natural compounds in ameliorating hepatic lipid accumulation by modulating mitophagy pathways. For example, cyanidin-3-O-glucoside has been shown to reduce oxidative stress, inhibit NLRP3 activation, and alleviate steatosis in HFD-induced models by upregulating PINK1–Parkin expression and enhancing their mitochondrial localization, thereby promoting PINK1-mediated mitophagy. Conversely, PINK1 knockdown attenuated the hepatoprotective effects of cyanidin-3-O-glucoside (41). Similarly, quercetin has been reported to activate the AMP-activated protein kinase signaling pathway, enhancing mitophagy, and reduce lipid accumulation under high-fat conditions (42). While current research has predominantly focused on the interplay between hepatic lipid accumulation and mitophagy in HFD-induced metabolic disorders, the specific regulatory role of mitophagy in HUA-induced hepatic lipid deposition remains largely unexplored.

Based on our previous work demonstrating that HUA activates hepatocyte autophagy through the reactive oxygen species–AMP-activated protein kinase–target of rapamycin pathway to alleviate hepatic insulin resistance (24), this study further elucidates the role of mitophagy in HUA-driven hepatic steatosis. Our experimental data reveal that HUA stimulation induces mitochondrial damage and activates mitophagy as an early protective response. However, we observed a temporal dissociation between mitophagy activation and lipid accumulation—while mitophagy markers peak at earlier time points, significant lipid accumulation manifests later at 48 h. This pattern suggests that mitophagy serves as a transient compensatory mechanism that may become attenuated under sustained metabolic stress.

The differential patterns of mitochondrial content regulation observed between acute cellular models and chronic animal models may offer valuable insights into the dynamic regulation of mitochondrial quality control under hyperuricemic conditions. In acute cellular settings, the coordinated activation of mitophagy along with enhanced mitochondrial biogenesis appears to maintain mitochondrial homeostasis, potentially representing a compensated adaptive state. In contrast, chronic animal models present a scenario where persistent mitophagy activation appears to coincide with reduced mitochondrial content, potentially indicating a transition toward decompensated mitochondrial quality control. We speculate that both mitophagy and biogenesis systems may experience functional overload under these conditions, which could disrupt the equilibrium between mitochondrial removal and regeneration. Furthermore, the complexity of the *in vivo* environment—characterized by systemic metabolic disturbances, interorgan crosstalk, and prolonged exposure to pathological stimuli—likely contributes to this overwhelmed state of mitochondrial quality control. These factors collectively might lead to a progressive impairment of the mitochondrial maintenance system, where autophagic initiation becomes increasingly uncoupled from degradation processes, ultimately resulting in net mitochondrial loss despite ongoing quality control efforts.

Notably, mitophagy inhibition through PINK1 knockdown upregulated CD36 expression and exacerbated lipid accumulation, underscoring mitophagy's protective role in HUA-induced metabolic dysregulation. While our finding of HUA-activated mitophagy contrasts with reports of mitophagy suppression in HFD-induced steatosis models, both scenarios demonstrate mitophagy's antagonistic role against lipid accumulation. We propose that HUA represents a reversible stressor that activates adaptive mitophagy, whereas HFD-induced persistent lipotoxicity (43), chronic inflammation (44), and lysosomal acidification dysfunction (45) ultimately lead to autophagic system collapse. This distinction highlights the context-dependent nature of mitochondrial adaptive responses and provides novel perspectives for understanding metabolic disease heterogeneity.

In summary, this study provides novel experimental evidence elucidating the relationship between hyperuricemia (HUA) and hepatic mitophagy, as well as the regulatory role of mitophagy in HUA-induced hepatic lipid accumulation. Our findings demonstrate that elevated UA levels upregulate CD36 expression, promoting hepatic lipid deposition, while concurrently activating the PINK1–Parkin signaling pathway to induce mitophagy. Notably, this mitophagy activation functions as a crucial protective mechanism and a compensatory response that counteracts HUA-induced hepatic steatosis through negative feedback regulation (Fig. 8). These results offer important insights into the dual regulatory roles of hyperuricemia in hepatic lipid metabolism and mitochondrial quality control. Based on these findings, we propose that targeted modulation of mitophagy may represent a promising therapeutic strategy for mitigating HUA-associated hepatic metabolic disorders. However, further research is warranted to gain a more comprehensive understanding of the molecular network linking mitophagy and hepatic lipid metabolism—particularly, the interplay between mitophagy and the CD36 signaling pathway.

## Experimental procedures

### Animal models and treatments

Male C57BL/6 mice (8 weeks old, 22–25 g) were obtained from the Animal Center of Xiamen University. *Uox* gene–deficient (*Uox*-KO) mice, a stable hyperuricemia model, were generated using the CRISPR–Cas9 genome-editing technique, as previously described in our work (33). Ten-week-old male WT and *Uox*-KO mice were randomly assigned to treatment or placebo groups (n = 6–8 per group). Mice received either the mitophagy activator Uro-A (50 mg/kg/day) or vehicle control (0.5% sodium carboxymethylcellulose) *via* oral gavage for 8 weeks.

At the experimental endpoint (week 18), mice were fasted overnight, and peripheral blood samples were collected from the orbital venous sinus. Euthanasia was performed *via* cervical dislocation. Fresh liver tissues were harvested, and the hearts were perfused with PBS followed by fixation with 4% paraformaldehyde. Liver tissues were subsequently dehydrated and fixed for further analysis. Blood samples were collected in 1.5 ml Eppendorf tubes, and serum was isolated by centrifugation at 3000 rpm for 15 min at 4 °C.

All animal experiments were conducted in accordance with protocols approved by the Xiamen University Animal Care and Use Committee (approval no.: XMULAC20230204). Throughout the study, except during fasting periods, animals had ad libitum access to food and water. Housing conditions were maintained to align with natural biological rhythms.

### Chemicals and reagents

Dulbecco's modified Eagle's medium, fetal bovine serum, and antibiotics were obtained from Gibco. Antibodies for PINK1 (1:1000 dilution; catalog no.: 6946), P62 (1:1000 dilution; catalog no.: 5114), and F4/80 (1:200 dilution; catalog no.: 70076) were sourced from Cell Signaling Technology; P-Parkin^S65^ (1:1000 dilution; Ab315376) was from Abcam; P-PINK1^S228^ (1:1000 dilution; AF7081) was from Affinity; TOM20 (1:200 dilution; catalog no.: 66777), Parkin (1:2000 dilution; catalog no.: 14060), ubiquitin (1:2000 dilution; catalog no.: 10201), and GAPDH (1:2000 dilution; catalog no.: 60004) from Proteintech, CD36 (1:1000dilution; NB400-144) from Novus Biologicals; and MFN2 (1:1000 dilution; A19678), LC3B (1:1000 dilution; A19665), and COXIV (1:1000 dilution; A11631) from Abclone. UA (U2625) was purchased from Sigma–Aldrich; MG132 (HY-13259) was purchased from MCE; and Uro-A (U287598) from Aladdin.

### Isolation and purification of primary mouse hepatocytes

Primary hepatocytes were isolated from 8- to 12-week-old male mice using a two-step collagenase perfusion method. Mice were anesthetized by isoflurane, and a needle was inserted into the hepatic portal vein for perfusion. The liver was first flushed with EDTA–PBS and then digested with collagenase IV. After dissection, hepatocytes were released, filtered, and centrifuged for purification. Viable cells were resuspended in attachment medium, assessed for viability, and cultured on collagen I-coated dishes. Cells adhered for 4 to 6 h before the medium was replaced for experimental use. Cellmatrix type I-A (631-00651) was sourced from Nitta Gelatin.

### Cell cultures

The human hepatoblastoma cell line (HepG2) was obtained from the Chinese Academy of Sciences. HepG2 cells were maintained in Dulbecco's modified Eagle's medium supplemented with 10% fetal bovine serum, 100 IU/ml penicillin, and 100 μg/ml streptomycin. Cells were cultured at 37 °C in a humidified atmosphere containing 5% CO_2_. The cell line was subjected to short tandem repeat authentication.

### Isolation of mitochondria from cells

Mitochondrial fractions from HepG2 cells were isolated using a Mitochondrial Isolation Kit (SM0020; Solarbio). The cells were collected and resuspended in 1 to 2 ml of ice-cold lysis buffer containing protease inhibitors and phosphatase inhibitors, followed by incubation on ice for 10 min. Cell membranes were disrupted by repeated shearing through a 1-ml syringe—approximately 30 passages for HepG2 cells—with over 60% membrane permeability confirmed by Trypan Blue staining. The lysate was centrifuged at 1000*g* for 5 min, and the supernatant was collected. This centrifugation step was repeated once to remove intact cells and nuclei. The supernatant was then centrifuged at 3000*g* for 10 min to pellet the mitochondria, whereas the resulting supernatant containing cytosolic proteins was removed. The mitochondrial pellet was resuspended in 0.5 ml of wash buffer and centrifuged at 1000*g* for 5 min. The supernatant from this step was collected and centrifuged again at 3000*g* for 10 min to obtain the purified mitochondrial pellet. After discarding the supernatant, the pellet was resuspended in 100 μl of radioimmunoprecipitation assay buffer and incubated for 10 min on ice to lyse mitochondrial proteins. Protein concentrations were measured using a bicinchoninic acid (BCA) assay. Finally, an appropriate volume of 5X loading buffer was added, and protein samples were boiled to denature the protein and stored at −80°C until use.

To assess the purity of the isolated mitochondrial proteins, we performed WB analysis comparing mitochondrial proteins with whole-cell proteins of equal protein content. The purity was evaluated by calculating the ratio of markers for cytosol and other organelles to mitochondrial markers (Fig. S1).

### Measurement of MMP

MMP was assessed using the Mitochondrial Membrane Potential Assay Kit with JC-1 (C2006; Beyotime). HepG2 cells were treated with 15 mg/dl UA for 48 h and incubated with the JC-1 probe as per the manufacturer’s instructions. And laser confocal microscopy (FV1000 MPE-B, 495; Olympus) was adopted to view the staining. The quantification of fluorescence values was quantified using ImageJ (NIH).

### Biochemical measurements

UA (C012), glucose (A154), TC (A111), TG (A110), low-density lipoprotein cholesterol (A113), high-density lipoprotein cholesterol (A112), ALT (C009), and aspartate aminotransferase (C010) levels were measured using commercial assay kits from Nanjing Jiancheng Bioengineering Institute. Serum, tissue, and cellular samples were processed according to the manufacturer's protocols. Protein concentrations in tissue and cellular samples were normalized for accurate comparative analysis.

### Histological staining

Tissue specimens were paraffin embedded and sectioned into 5-μm thick slices. H&E staining was performed following standard protocol. For Oil Red O staining, 10-μm frozen liver sections and cell climbing slices were stained to assess lipid accumulation. Stained slides were imaged using a VM1 Digital Slide Scanning System.

### WB analysis

Cells and tissues were lysed in radioimmunoprecipitation assay buffer with protease and phosphatase inhibitors, and protein concentrations were measured using a BCA assay. Proteins (20–50 μg) were separated by SDS-PAGE (10% or 12%) and transferred to polyvinylidene fluoride membranes. Membranes were blocked with 5% skim milk, incubated overnight with primary antibodies, and then with horseradish peroxidase–conjugated secondary antibodies (1:2000–1:5000 dilution, 31430 [mouse], 31460 [rabbit]; Thermo Fisher). Bands were detected using SuperKine West Femto substrate and visualized with a Chemi Scope 6200 Touch system. ImageJ was used for quantification, with GAPDH as a loading control.

To ensure the suitability of GAPDH as a loading control under our experimental conditions, we performed systematic validation in both cellular and animal models. The expression stability of two housekeeping genes (GAPDH and ACTIN) was simultaneously examined, supplemented by Ponceau S staining as an additional reference (Fig. S2).

### RNA isolation and qPCR

Total RNA was extracted from liver tissues and hepatocytes using Trizol reagent. Reverse transcription was performed with 1.0 μg total RNA using a RevertAid Master Mix (M16315; Thermo Fisher). Primer sequences (Table S1) were designed using the PrimerBank database and validated *via* BLAST. qPCR was conducted using Hieff qPCR SYBR Green Master Mix (11200ES; Yeasen) in a 20 μl reaction volume. Gene expression levels were normalized to GAPDH.

### RNA interference

siRNA targeting PINK1 (si-PINK1) and CD36 (si-CD36) were synthesized by Qingke Biotechnology. The siRNA sequences were si-PINK1: sense: 5′-GCACACUGUUCCUCGUUAUTT-3′, antisense: 5′-AUAACGAGGAACAGUGUGCTT-3′; si-CD36: sense: 5′-GCUAUUGCGACAUGAUUAATT-3′, antisense: 5′-UUAAUCAUGUCGCAAUAGCTT-3′. Transfection was performed using Lipofectamine 2000 (18324012; Thermo Fisher) following the manufacturer’s protocol. Briefly, 5 μl of Lipofectamine 2000 and 5 μl of siRNA were separately diluted in 250 μl of Opti-MEM (31985062; Thermo Fisher) and incubated for 5 min at room temperature. The solutions were then combined, gently mixed, and incubated for 20 min to form siRNA–lipid complexes. These complexes were added to hepatocytes cultured in 6-well plates, and downstream experiments were conducted 6 h post-transfection.

### Immunohistochemistry

Paraffin-embedded tissue sections were deparaffinized, rehydrated, and subjected to antigen retrieval in sodium citrate buffer. Endogenous peroxidase activity was blocked using 3% hydrogen peroxide–methanol solution. Sections were incubated with 10% goat serum for 1 h at room temperature to block nonspecific binding, followed by overnight incubation at 4 °C with anti-F4/80 monoclonal antibody (1:200 dilution). Horseradish peroxidase–conjugated secondary antibodies (1:200 dilution) were applied for 1 h at room temperature, and immunoreactivity was detected using a DAB chromogen kit.

### Immunofluorescence

Primary hepatocytes were seeded into confocal dishes and treated with 15 mg/dl UA for 9 h. Cells were washed with PBS and fixed with ice-cold methanol for 20 min at 4 °C. After blocking with 10% goat serum for 1 h at room temperature, cells were incubated overnight at 4 °C with primary antibodies against LC3B (1:200 dilution) and TOM20 (1:200 dilution). After washing, cells were incubated with Alexa Fluor 594–conjugated goat anti-rabbit IgG (1:200 dilution, A11011; Thermo Fisher) and Alexa Fluor 488–conjugated goat anti-mouse IgG (1:200 dilution, 4408; Cell Signaling Technology) for 1 h at room temperature. Fluorescence images were captured using a Nikon A1R confocal microscope (Nikon) and analyzed with ImageJ.

### Lipid droplet fluorescence staining

The lipid droplet in primary hepatocytes was stained using a Lipid Droplets Green Fluorescence Assay Kit with BODIPY 493/503 (C2053; Beyotime). Primary mouse hepatocytes were seeded on cell climbing slices and treated with 15 mg/dl UA for 48 h, followed by lipid droplet fluorescence staining according to the manufacturer's instructions. Fluorescence images were captured using a Nikon A1R confocal microscope (Nikon) and analyzed with ImageJ.

### ATP content determination

The ATP content in primary hepatocytes was measured using a Chemiluminescence Assay Kit (E-BC-F002; Elabscience). Primary mouse hepatocytes were treated with 15 mg/dl UA for 48 h, followed by ATP content determination according to the manufacturer's instructions. Luminescence intensity was recorded using a microplate reader (Varioskan Flash; Thermo). The ATP concentrations in the samples were calculated based on their comparison with a standard curve. The ATP levels were adjusted based on the total protein content measured by the BCA method, and the relative ATP content for each group was calculated accordingly.

### Transmission electron microscopy

Mouse primary hepatocytes were cultured on collagen I-coated 60-mm dishes and treated with 15 mg/dl UA for 48 h. Cells were washed with PBS and fixed with 2.5% glutaraldehyde in PB buffer at room temperature for 5 min, then transferred to 4 °C for 2 to 3 h. Samples were washed three times with PB buffer and incubated in PB buffer containing 5% BSA. Cells were gently scraped, transferred to 1.5 ml centrifuge tubes, and centrifuged at 2500*g* for 5 min at 4 °C. The resulting cell pellets were submitted to the Xiamen University Biomedical Instrumentation Sharing Platform for further processing and electron microscopy imaging.

### RNA sequencing

Liver tissues were collected from 12-week-old WT and *Uox*-KO mice, followed by total RNA extraction. RNA-Seq was carried out by Metware on the Illumina platform to generate 150 bp paired-end reads. Briefly, total RNA was extracted, and libraries were constructed following mRNA enrichment, fragmentation, and complementary DNA synthesis. After sequencing, raw reads were quality-filtered using fastp and aligned to the reference genome using HISAT2. Gene expression was quantified, and differential expression analysis was conducted with DESeq2. Functional enrichment analysis was performed for Gene Ontology and Kyoto Encyclopedia of Genes and Genomes terms.

### Statistical analysis

Statistical analyses were performed using GraphPad Prism (version 10.1.2; GraphPad Software, Inc). Data are presented as mean ± SD. Comparisons between two groups were conducted using unpaired Student’s *t* tests, whereas comparisons among multiple groups were evaluated using one-way analysis of variance or two-way analysis of variance. *p* < 0.05 was considered statistically significant.

## Data availability

Data supporting the findings in the article are available from the corresponding author upon request. The RNA-Seq data have been deposited into the EMBL-EBI with the accession number E-MTAB-16271.

## Supporting information

This article contains supporting information.

## Conflict of interest

The authors declare that they have no conflicts of interest with the contents of this article.
