# Supplementary material for: Activation of mitophagy antagonizes high uric acid–induced hepatic lipid accumulation
Source: J Biol Chem. 2025 Dec 13;302(2):111054. doi: 10.1016/j.jbc.2025.111054 (PMC12805366; doi:10.1016/j.jbc.2025.111054)

**Supplementary Table 1.** Primer sequence

| **Gene** | **Gene ID** | **Forward Sequence** | **Reverse Sequence** |
| --- | --- | --- | --- |
| Pink1 | 68943 | TTCTTCCGCCAGTCGGTAG | CTGCTTCTCCTCGATCAGCC |
| Prkn | 50873 | GAGGTCCAGCAGTTAAACCCA | CACACTGAACTCGGAGCTTTC |
| Sqstm1 | 18412 | TGTGGAACATGGAGGGAAGAG | TGTGCCTGTGCTGGAACTTTC |
| Bnip3 | 12176 | TCCTGGGTAGAACTGCACTTC | GCTGGGCATCCAACAGTATTT |
| Fundc1 | 72018 | CCCCCTCCCCAAGACTATGAA | CCACCCATTACAATCTGAGTAGC |
| Mfn1 | 67414 | GCAGACAGCACATGGAGAGA | GATCCGATTCCGAGCTTCCG |
| Mfn2 | 170731 | GGTCAGGGGTATCAGCGAAG | TTGTCCCAGAGCATGGCATT |
| Opa1 | 74143 | AACAGCATTTCGAGCAACAGA | CTTCCGCAGCTCTTTGTTCTC |
| Dnm1l | 74006 | CCCGGAGACCTCTCATTCTG | GTCTTGAGTTTTTCCATGTGGC |
| Vdac1 | 22333 | CCCACATACGCCGATCTTGG | GTGGTTTCCGTGTTGGCAGA |
| Ppargc1a | 19017 | TATGGAGTGACATAGAGTGTGCT | CCACTTCAATCCACCCAGAAAG |
| Nrf1 | 18181 | CGGAAACGGCCTCATGTGT | CGCGTCGTGTACTCATCCAA |
| Tfam | 21780 | CAAAGGATGATTCGGCTCAGG | TCGACGGATGAGATCACTTCG |
| Srebf1 | 20787 | GTGAACATCTCCTAGAGCGA | CTGAGAACTCCCTGTCTCC |
| Acaca | 107476 | TCCGTCAGCTCAGATACAC | GACATGCTGGATCTCATGTG |
| Fasn | 14104 | CTGACTCGGCTACTGACAC | TTAGGGTAGGACCCTCAGG |
| Scd1 | 20249 | TGCTCCAAGAGATCTCCAG | GTCTTCTTCCAGGTGGAGG |
| Cpt1a | 12894 | CTCCGCCTGAGCCATGAAG | CACCAGTGATGATGCCATTCT |
| Acox1 | 11430 | GAAATATGCCCAGGTGAAGC | CACGAGGAAGGACCTTACG |
| Ppara | 19013 | AATTTGCTGTGGAGATCGG | TTAAGCACGTGCACAATCC |
| Pparg | 19016 | GATGTCTCACAATGCCATCAG | ATATCACTGGAGATCTCCGC |
| Slc27a2 | 26458 | TGCAGGAAATACAACGTCAC | ATTTGGTTTCTGCGGTGTG |
| Slc27a5 | 26459 | TACAAGTTGGAGCCACCTG | ATCACTGTTACGCCATGCT |
| Cd36 | 12491 | CACATACAGAGTTCGTTATCTAGC | CAAAGATGGCTCCATTGGG |
| Fabp1 | 14080 | ATTCATGAAGGCAATAGGTCTG | TCATGCACGATTTCTGACAC |
| Mttp | 17777 | CTTCATGGTAGTCAGGTGG | GTTCTCCTCTCCTTCATCAG |
| Gapdh | 14433 | AGGTCGGTGTGAACGGATTTG | TGTAGACCATGTAGTTGAGGTCA |


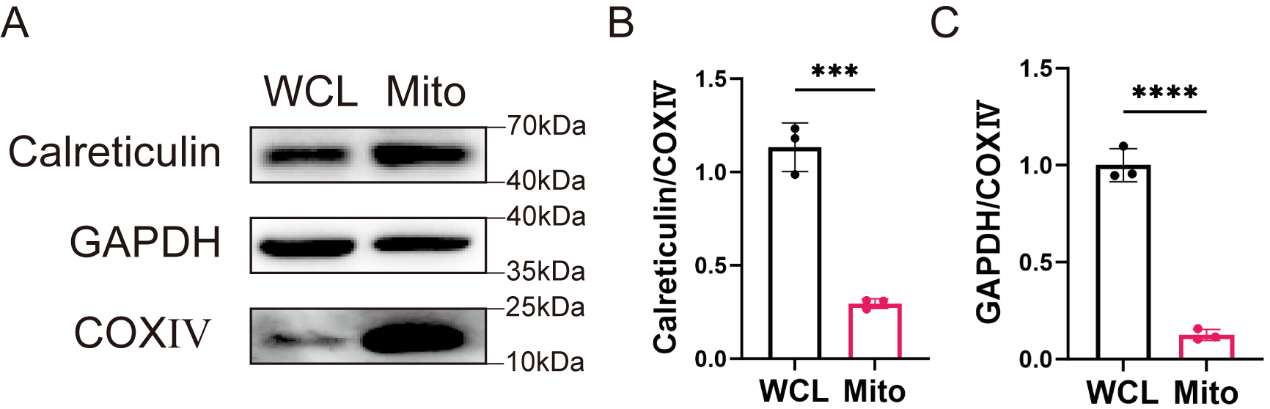
**Figure S1. Mitochondrial Fraction Purity Validation.** **(A)** Representative western blot images of Calreticulin (ER), GAPDH (cytosol), and COXⅣ (mitochondrial) protein expression in whole-cell lysates (WCL) and mitochondrial fractions. **(B-C)** Quantification of Calreticulin/COXⅣ and GAPDH/COXⅣ levels in WCL and mitochondria fractions (n = 3).

**Figure S2. Validation of GAPDH as a loading control in cellular and animal models. (A)** Western blot analysis of GAPDH and ACTIN expression, along with Ponceau S staining, in mouse primary hepatocytes treated with 15 mg/dL uric acid for varying time periods. The GAPDH panel in (A) is the same as that shown in Figure 3A. **(B)** Western blot analysis of GAPDH and ACTIN expression, together with Ponceau S staining, in liver tissues from WT and *Uox*-KO mice. The GAPDH panel in (B) is the same as that shown in Figure 4A.
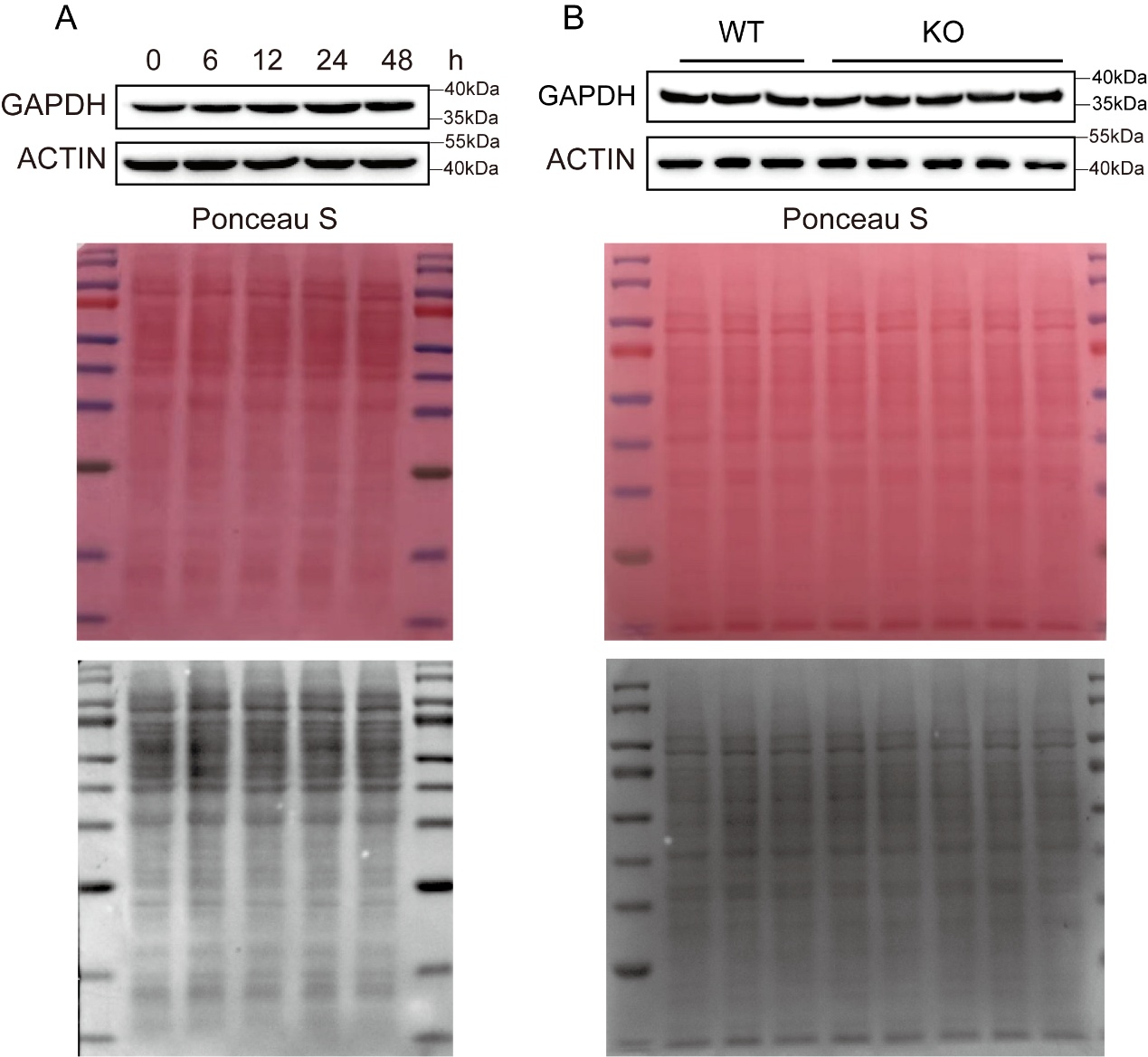

Supplement: Supplementary Material 1 [file mmc1.docx]
